# A rapid, automated surface protein profiling of single circulating exosomes in human blood

**DOI:** 10.1038/srep36502

**Published:** 2016-11-07

**Authors:** Golam Kibria, Erika K. Ramos, Katelyn E. Lee, Sarah Bedoyan, Simo Huang, Ravand Samaeekia, Jaffre J. Athman, Clifford V. Harding, Jan Lötvall, Lyndsay Harris, Cheryl L. Thompson, Huiping Liu

**Affiliations:** 1 Department of Pathology, School of Medicine, Case Western Reserve University, Cleveland, Ohio, USA; 2 The Institute Molecular Engineering, the University of Chicago, Chicago, Illinois, USA; 3 Krefting Research Centre, Department of Internal Medicine and Clinical Nutrition, University of Gothenburg, Gothenburg, Sweden; 4 Department of Medicine, School of Medicine, Case Western Reserve University, Cleveland, Ohio, USA; 5 Department of Nutrition, School of Medicine, Case Western Reserve University, Cleveland, Ohio, USA; 6 The Case Comprehensive Cancer Center, Cleveland, Ohio, USA; 7 The National Center for Regenerative Medicine, Case Western Reserve University, Cleveland, Ohio, USA; 8 Department of Pharmacology, Feinberg School of Medicine, Northwestern University, Chicago, Illinois, USA.

## Abstract

Circulating exosomes provide a promising approach to assess novel and dynamic biomarkers in human disease, due to their stability, accessibility and representation of molecules from source cells. However, this potential has been stymied by lack of approaches for molecular profiling of individual exosomes, which have a diameter of 30–150 nm. Here we report a rapid analysis approach to evaluate heterogeneous surface protein expression in single circulating exosomes from human blood. Our studies show a differential CD47 expression in blood-derived individual circulating exosomes that is correlated with breast cancer status, demonstrating a great potential of individual exosome profiles in biomarker discovery. The sensitive and high throughput platform of single exosome analysis can also be applied to characterizing exosomes derived from other patient fluids.

Exosomes are cell-secreted extracellular vesicles (EVs) between 30–150 nm in size with a closed double-layer membrane structure^1^,^2^,^3^. They exist in virtually all body fluids and carry various molecules (proteins, lipids, and RNAs) on their surface as well as in the lumen^1^,^2^,^3^. Exosomes play a critical role in intercellular communication and cellular content transfer, e.g. mRNAs and microRNAs, in both physiological and pathological settings, such as tumor progression^4^,^5^,^6^,^7^. The exosomal surface proteins can mediate organ-specific homing of circulating exosomes, and their contents show potential to serve as novel biomarkers^8^,^9^,^10^, thereby assisting the diagnosis and prognosis of human diseases, such as cancer. Further, analyzing the dynamic changes of the exosome contents may provide a way to monitor disease.

Approaches to exosome characterization include: (1) electron microscopy (EM) to assess structure and size; (2) nanoparticle tracking analysis (NTA)^3^ to reveal size and zeta potential; (3) protein analysis via immunofluorescence staining, western blotting, ELISA, and mass spectrometry, (4) RNA analysis using array platforms, RNA sequencing, and PCR, and (5) analysis of lipids, sugar, and other components by biochemical assays. Among these approaches, EM provides high-resolution imaging but is neither convenient nor affordable for high throughput molecular profiling of large numbers of circulating exosome samples for potential clinical applications. NTA utilizes light scattering and Brownian motion^3^ to measure particle size but does not differentiate between vesicles within a size range of 5 orders of magnitude due to the low dynamic range of the camera^11^. In addition, NTA is not suitable for molecular profiling of exosomes because of low sensitivity to fluorescent signals. While RNAs and lipids also potentially serve as molecular biomarkers of circulating exosomes in human disease, there is a need to improve protein profiling of exosomes, as oftentimes the protein expression is the most clinically relevant marker.

Since exosomes are heterogeneous, and only a subset of circulating exosomes may express a specific biomarker molecule of interest, our study sought to develop a feasible approach for rapid and high throughput profiling of surface proteins at a single exosome level, a major challenge to moving the field of exosome-based biomarkers forward. Flow cytometry is a commonly used optical method to analyze cells based on the light scattering and fluorescence-activated mechanisms. However, conventional flow cytometers have a minimum detection size of 200–500 nm that is beyond the size of exosomes, and they are ineffective at discriminating particles that differ by 100–200 nm or less^2^,^12^. In conventional flow cytometry, the background signal is often high, in the <200 nm size range, due to contaminating particles in the sheath buffer. Furthermore, the level of immunolabeling signal is limiting in such small particles. Recently, latex beads in micrometer sizes have been used to bind to multiple exosomes to enhance the ability to detect exosomes stained with fluorophore-conjugated antibodies by conventional flow cytometry^10^. However, this bead-based approach does not provide single exosome profiling and therefore fails to discriminate between different subsets of exosomes, which may result in the loss of distinctive signatures with potential diagnostic importance.

Here we report a new, automated analytic approach utilizing a micro flow cytometer^13^, and present data on its use to profile protein expressions of individual exosomes isolated from cell lines and human blood of breast cancer patients and healthy controls, as a proof of principle. We first assessed the expression of an exosomal marker, CD63, in cell-line derived exosomes following a rapid staining preparation and automated reading/counting procedure. Then we expanded to measure two cancer-related surface proteins, CD44^14^,^15^,^16^,^17^,^18^,^19^ and CD47^20^,^21^,^22^,^23^,^24^ in human blood-derived exosome specimens to assess correlations of these markers on exosomes with cancer status^14^. CD44 is a known marker for breast tumor initiating cells and is involved in tumor progression^14^,^15^,^16^,^17^,^18^,^19^. The expression of CD47 on the surface of the cancer cells prevents recognition by macrophages and natural killers, thereby inhibiting their ability to engulf and destroy those cancer cells^25^,^26^.

## Results

**Figure 1:**
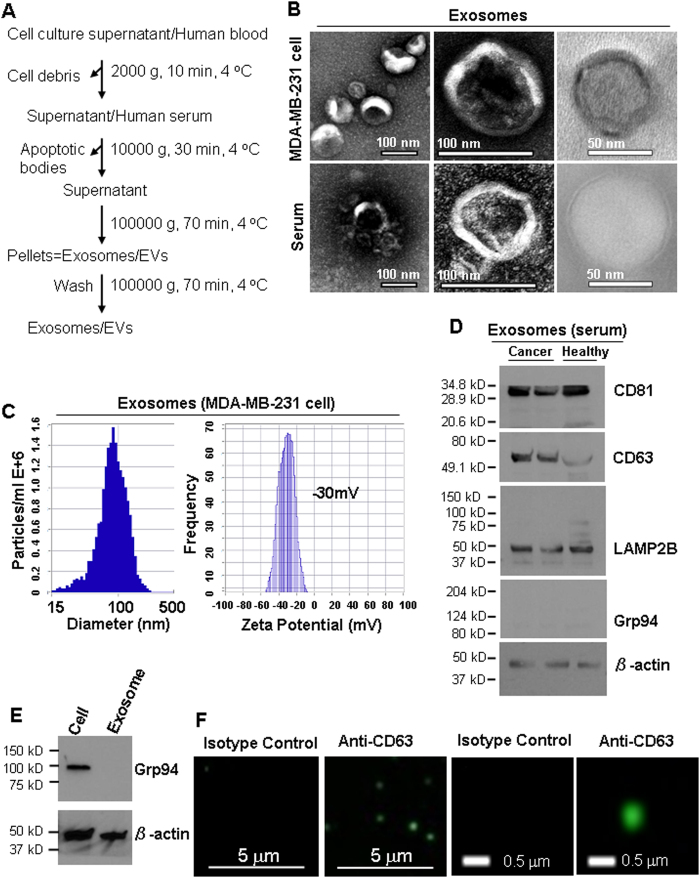
Isolation and characterization of exosomes from cell culture supernatant and human blood. (**A)** Exosome purification procedure by ultracentrifugation. Blood was centrifuged at 3200 RPM for 15 min at 4 °C to collect serum. As mentioned in the first step, the cell culture supernatant was centrifuged at 2000 g, 10 min, 4 °C to remove cell debris. (**B**) Observation of the morphology of exosomes under Transmission Electron Microscopy (TEM), indicating the diameter of isolated exosomes in 50–100 nm. (**C**) ZetaView NTA analysis of MDA-MB-231 cell-derived exosomes with the size distribution (mean diameter 89 ± 33 nm and mode 87 nm) and surface charge (−30 mV). (**D**) Immunoblot of exosomal markers CD81 (~30 kDa), CD63 (~55 kDa), and LAMP2B (~50 kDa) in exosomes (5 μg lysates) isolated from the serum of breast cancer patients and healthy control. Grp94 (~100 kDa) and β-actin (~42 kDa) serve as a negative control and a loading control, respectively. (**E**) Immunoblot of Grp94 with 5 μg protein of MDA-MB-231 cell lysates (Cell) and the exosomes derived from these cells (Exosome, no Grp94 detection). (**F**) Detection of CD63 in intact MDA-MB-231 exosomes by immunofluorescence staining.

Exosomes from breast cancer MDA-MB-231 cells and human serum samples were mainly isolated by differential ultracentrifugation^27^ (Fig. 1A) unless specified in this report, which remains the most widely used and unbiased purification method^28^. In addition to differential ultracentrifugation method, we also isolated exosomes following a different method using the exosome-isolation kit from Thermo Fisher. Both methods consistently purified exosomes with a double-layer membrane structure and a size range of 50–100 nm as observed by TEM (Fig. 1B, Supplementary Fig. S1). According to the guidelines of the International Society of Extracellular Vesicles (ISEV) for the characterization of exosomes^29^, multiple approaches were used to characterize the physical features and molecular markers of the isolated extracellular vesicles in order to identify these as exosomes. Measured by NTA (ZetaView), the mean size of exosomes was 89 ± 33 nm and the surface charge of exosomes was about −30 mV (Fig. 1C), indicating the presence of negatively charged molecules on the surface of exosomes. Immunoblotting and immunofluorescence staining analyses of purified exosomes (without bead conjugation) confirmed the presence of at least three exosomal markers such as CD63, CD81 and LAMP2B^28^,^30^, as well as the absence of Grp94 expression in the exosomes isolated from the serum (Fig. 1D) and cultured cells (Fig. 1E,F, Supplementary Fig. S2). Additionally, the presence of CD81 and LAMP2B, as well as the absence of Grp94 markers were also observed in the exosomes isolated from human serum using the exosome-isolation kit (Supplementary Fig. S3).

**Figure 2:**
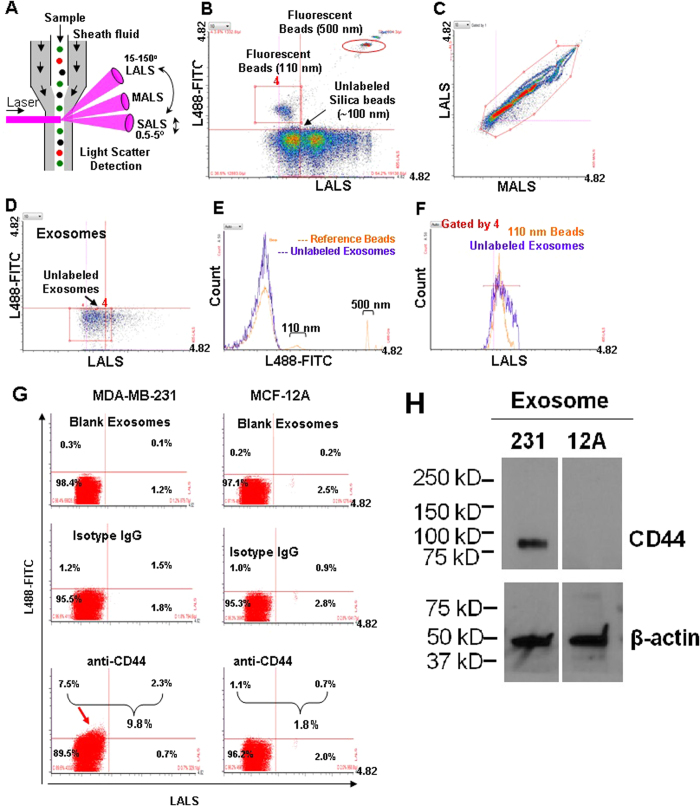
MFC detection of the ApogeeMix Beads and blood exosomes. (**A**) Schematic representation of Apogee A50 MFC denoting the mode of action of nanoparticle detection. The sample flows from top to bottom and is surrounded by sheath fluid. The laser intersects with the sample stream, generating 3 different light scatters (LALS, MALS, SALS) and fluorescence signals. LALS: Large angle light scatter; MALS: Middle angle light scatter; SALS: Small angle light scatter. (**B**) Cytogram of the reference beads, an aqueous mixture of the green fluorescent latex and non-fluorescent silica (Si) spheres in 110–1300 nm plotted at green fluorescence (L488-FITC) and LALS signals with minimized background noise. The red rectangle and circled box represent the 110 nm and 500 fluorescence beads respectively. (**C**) Cytogram of the reference ApogeeMix Beads in 110–1300 nm with similar MALS and LALS signals at the modified high threshold setting. (**D**) Cytogram of the unlabeled exosomes from human blood at green fluorescence and LALS signals. (**E**) Fluorescent signal comparison between the ApogeeMix beads from B and the unlabeled exosomes from D shown at the L488–FITC channel. (**F**) Histogram comparing the size of the gated exosomes from D with that of the gated 110 nm fluorescence beads in B (red rectangles). (**G**) Flow analysis of CD44 expression on exosomes derived from MDA-MB-231 and MCF-12A cells using A50 MFC. (**H**) Immunoblot of CD44 expression in the exosome lysates derived from MDA-MB-231 and MCF-12A cells. β-actin (~42 kDa) serves as a loading control.

To profile individual circulating exosomes, we utilized the Apogee A50 Micro flow cytometer (MFC) that detects smaller particles with three light scatters: small angle light scatter (SALS), middle angle light scatter (MALS), and large angle light scatter (LALS)^31^ (Fig. 2A) as well as fluorescent channels based on the laser(s) of choice. To minimize the background noise of PBS and the reference ApogeeMix beads shown at the default setting (Supplementary Fig. S4A and the purple box in S4B), we then modified the settings to a higher threshold to run PBS alone (Supplementary Fig. S4C) and the beads (Fig. 2B,C). We then evaluated the fluorescence and size features of the blood-derived unlabeled circulating exosomes using L488-FITC and LALS signals (Fig. 2D,E). Based on the comparison histograms in Fig. 2F, the size curve of the gated circulating exosomes in Fig. 2D (red rectangle) was found close to or largely overlapping with that of the 110 nm fluorescence beads gated in Fig. 2B (red rectangle).

Using fluorophore-conjugated antibodies, we set out to measure expression levels of the exosomal surface marker CD63 and other cancer-related proteins shown in our mass spectrometry analyses of exosomes, such as CD44 and CD47. Prior to incubation or staining with the exosomes, the antibody solutions were centrifuged to eliminate any existing background particles (Supplementary Fig. S4D). CD63 expression was detectable by MFC in the cell line-derived exosomes that served as a positive control (Supplementary Fig. S5A). The differential expression of CD44 was detected by A50 MFC on the exosomes derived from MDA-MB-231 cells (mainly CD44 positive) and MCF-12A cells (mainly CD44-negative) (Fig. 2G), and consistently validated by CD44 immunoblotting of these exosomes (Fig. 2H).

We then optimized the detection procedure to measure CD47 and CD44 levels in the circulating exosomes from human blood (cancer patients n = 60 and healthy controls n = 60). A summary of the clinical characteristics of the breast cancer patients was provided in Supplementary Table S1. For each sample, an aliquot of purified exosomes (based on a total protein of 2 μg) was incubated with a specific antibody, its isotype control, or a blank buffer control for 45 min at 4 °C to avoid aggregations. Upon 25-fold volume dilution, the exosomes were then immediately analyzed on MFC (~4500 events collected).

**Figure 3:**
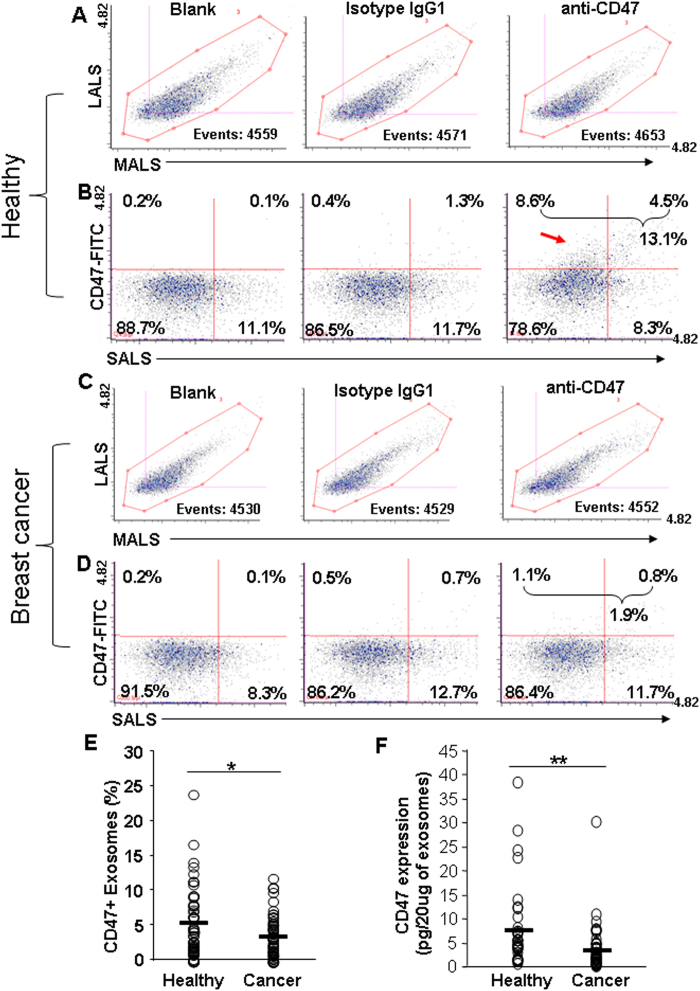
Detection of the surface protein CD47 in circulating exosomes and its correlation with breast cancer. (**A,C**) Representative scattered plots showing MALS and LALS signals of the circulating exosomes isolated from the blood of healthy control (**A**) and breast cancer patients (**C**), 4500 total events collected for each sample: unstained blank control, isotype IgG1, and anti-CD47. (**B,D**) Representative cytograms showing differential expression of CD47 on the exosomes isolated from the blood of healthy control (**B**) or that of a breast cancer patient (**D**). Exosomes were stained with FITC-CD47 antibody or isotype control FITC-IgG1, or unstained (control). (**E)** Comparison of MFC-analyzed CD47 expression levels on circulating exosome specimens isolated from the blood of healthy people (representative in **B**, n = 60) and breast cancer patients (representative in **D**, n = 60). Unpaired Student’s t-test, p value < 0.05. (**F**) Expression of CD47 on circulating exosomes measured by the ELISA. Exosomes isolated from the blood of 40 healthy control and 50 breast cancer patients were treated with the anti-CD47 ELISA antibody. Unpaired Student’s t-test, p value < 0.01.

The exosomes in the three staining conditions had similar profiles of MALS/LALS (Fig. 3A,C). From the healthy control samples, CD47 expression was remarkably detected in ~10% of individual circulating exosomes whereas minimal CD47 expression (0.7%, after deducting the background signal) was shown in the circulating exosomes from breast cancer patients (Fig. 3B,D). A significant difference of CD47 expression was observed between exosomes from cancer patients (n = 60) versus exosomes from healthy control (n = 60, p = 0.037) (Fig. 3E). The exosomal CD47 expression profiles were similar between the analyses from 5,000 and 10,000 collected exosome counts (Supplementary Fig. S5B). Furthermore, the expression of CD47 was detected on exosomes isolated from the human serum by using the exosome-isolation kit, but it required additional clean-up via ultracentrifugation in order to reduce the false-positive noise background for flow analyses (Supplementary Fig. S6B).

We further validated CD47 expression in exosomes using a second approach, CD47-ELISA which required a minimum of 20 μg proteins of collected exosomes. We also observed a significant difference of CD47 protein levels between two groups of exosome samples derived from healthy people and age-matched breast cancer patients (p = 0.004) (Fig. 3F). In contrast to the differential CD47 expression profiles, we did not observe a significant difference of CD44 protein levels between the exosomes from the healthy control group and those from the breast cancer patients (Supplementary Fig. S7A,B). These results suggest a great potential for single exosome profiling technology in discovering specific novel diagnostic biomarkers in cancer.

## Discussion

Due to improved ability to rapidly analyze small particles at an unprecedented sensitivity for fluorescent signals and extreme light scatter performance using three distinct angle ranges, the MFC is capable of measuring surface protein profiles of single exosomes isolated from cell culture or human blood. It may greatly improve high throughput, dynamic analyses of human body fluid-derived extracellular vesicles (such as exosomes and micro vesicles from blood and urine) and will expedite discoveries of novel diagnostic and prognostic biomarkers. The functional importance of differential CD47 expression detected in circulating exosomes from healthy versus cancer populations needs further study. The related molecular mechanisms contributing to this differential expression profile may involve differential rates of exosome production or exosome clearance.

In addition to proteins, nucleic acids and lipids in exosomes can also be analyzed after appropriate staining with suitable fluorescent reagents. Upon protocol optimization, it might also be possible to detect proteins in the lumen. In this study, we present a unique, sensitive, high throughput platform that can be applied to evaluate exosomes for clinical cancer diagnosis. Future studies can evaluate the potential of this method to characterize proteins in exosomes derived from other patient fluids, such as urine or saliva.

## Methods

### Human Studies

All human blood studies were performed in compliance with the US Department of Health and Human Services and approved by The University Hospitals Case Medical Center (UHCMC) Institutional Review Board CASE 9114 (IRB number 01–15–35C) “The role of exosomes in breast cancer”. Informed consent was obtained from all subjects when the blood was originally collected.

### Cell Culture

The human breast adenocarcinoma cells (MDA-MB-231) and breast epithelial cells (MCF-12A) were purchased from the American Type Culture Collection, ATCC (Manassas, VA, USA). Before culturing, the cells were tested for mycoplasma contamination. The cells were grown in Dulbecco’s Modified Eagle’s Medium (DMEM) supplemented with 5% (v/v) fetal bovine serum (FBS), 100 U/mL penicillin and 100 mg/mL streptomycin. MCF-12A cells were cultured in a mixture of DMEM and Ham’s F12 medium (1:1 v/v) with 20 ng/ml human epidermal growth factor, 100 ng/ml cholera toxin, 0.01 mg/ml bovine insulin, 500 ng/ml hydrocortisone and 5% horse serum (v/v). To prepare the complete medium for cell culture, FBS or horse serum was exosome-depleted by ultracentrifugation at 100,000 ×  *g* for 16 h at 4 °C.

### Isolation and Purification of Exosomes from Cells

Exosomes were isolated from the cell culture supernatant, as described, previously^1^. A scheme of the isolation protocol is given in Fig. 1A. Briefly, the cells were cultured as monolayers for 48 h in respective complete medium under an atmosphere of 5% CO_2_ at 37 °C. When cells reached a confluency of approximately 80% after 48 h, exosomes were isolated by differential centrifugation. First, the culture supernatant was centrifuged at 2,000 × *g* for 10 min followed by 30 min centrifugation at 10,000 × *g* to remove dead cells and cell debris. The clarified supernatant was ultracentrifuged for 2 h at 100,000 × *g* using an SW28 rotor to pellet the exosomes. Exosomes were washed by resuspension in 30 mL of sterile PBS (Hyclone, Utah, USA), and pelleted by ultracentrifugation for 2 h at 100,000 × *g*. The final exosome pellet was resuspended in 100 μl PBS and stored at −80 °C.

### Isolation and Purification of Exosomes from Human Blood

Serum was derived from human blood (of healthy volunteers and/or of breast cancer patients) by centrifugation using Thermo IEC Centra-GP8R Centrifuge (Artisan Technology Group, Champaign, IL, USA) at 3200 RPM for 15 min at 4 °C. Blood was drawn from healthy population or age and sex-matched breast cancer patients before they were treated. The samples were then aliquoted and frozen at −80 °C until needed. Serum (0.5 ml) was diluted to 4 ml with PBS and centrifuged at 15,000 × g for 30 min at 4 °C using TLA50.1 rotor to remove any remaining cells and debris followed by ultracentrifugation at 100000 × g for 2 h at 4 °C to pellet the exosomes. The exosomes were resuspended in 2 ml of PBS and further pelleted by ultracentrifugation for 2 h at 100,000 × *g*. Finally, the exosome pellet was resuspended in 50 μl of PBS and frozen at −80 °C.

For isolation of exosomes from serum using the exosome-isolation kit (Thermo Fisher), serum was centrifuged at 2000 × g for 30 minutes at 4 °C to remove the cell debris. The supernatant was collected and re-centrifuged at 10,000 × g for 30 minutes to remove the apoptotic bodies. The supernatant was transferred to a new tube and incubated with the exosome-isolation kit (Cat#4478360, Life Technologies, Carlsbad, CA, USA) for 30 min at 4 °C. After incubation, the mixture was centrifuged at 10,000 × g for 10 min at room temperature to get the exosome pellet. For further purification, exosomes were resuspended in 4 ml of PBS and pelleted by ultracentrifugation for 2 h at 100,000 × *g*. Finally, the pellet was resuspended in PBS and frozen at −80 °C.

### Electron Microscopy

Formvar/carbon-coated EM grids (Gilder Nickel Grid, Electron microscopy Sciences, PA, USA) were placed Formvar/carbon side down on top of the exosome sample drops for 10 minutes at room temperature. The grids were removed, blotted with filter paper and placed onto drops of freshly prepared 2.0% uranylacetate aqueous solution for one minute. The excess uranylacetate solution was removed, and air-dried. The images of the exosomes were captured using the FEI Tecnai™ Spirit (T12) transmission electron microscopes (Hillsboro, Oregon, USA) with a Gatan US4000 4kx4k charge coupled device (CCD) camera.

### Size Distribution and Zeta-potential of Exosomes

The size and Zeta-potential of the exosomes were measured using ZetaView® nanoparticle tracking analyzer (Particle Metrix GmbH, Meerbusch, Germany).

### Immunostaining

Exosomes were mixed with 2% PFA (1:1, v/v in PBS) and placed on a coverslip for 20 min, followed by washing twice with PBS. Exosomes were blocked with 10% Normal Donkey Serum (Cat# 017-000-121, Jackson Immunoresearch Labs Inc., West Grove, PA, USA) for 30 min and washed with PBS. Following this, exosomes were treated with the mouse anti-human CD63 antibody (Cat# ab8219, Abcam, Cambridge, MA, USA) at a concentration of 4 μg/ml for 20 min at room temperature and then washed with PBS. Finally, the exosomes were incubated with the Alexa 488 goat anti-mouse IgG (Cat#A11001, Invitrogen, Eugene, Oregon, USA) at a dilution of 1:200 for 20 min followed by washing with PBS, and fixed using ProLong® Gold Antifade Mountant (Cat# P10144, Carlsbad, CA, USA). The treated exosomes were observed under a confocal microscope (DeltaVision Elite, GE Healthcare Life Sciences, PA, USA). Exosomes those were either untreated or treated only with Alexa 488 goat anti-mouse IgG served as background controls.

### Rapid Micro Flow Cytometer Analysis of Exosomes

Before treating the exosomes with the antibodies, the antibody solutions were centrifuged at 14000 × g for 1 h at 4 °C to remove any aggregates. To detect surface proteins, exosomes (2 μg protein equivalent amount of exosomes in 20 μl of PBS) were blocked by using 1 μg of IgG from mouse serum (Cat# l5381, Sigma, St. Louis, MO, USA) for 10 min at 4 °C and incubated with 0.1 μg of FITC mouse anti-human CD47 (Cat#556045, BD Biosciences, San Jose, CA, USA) for 45 min at 4 °C. Consequently, the solution was diluted to 500 μL with PBS (Hyclone, Utah, USA). Finally, without any further washing, the samples were run on Apogee A50 Micro Flow Cytometer (MFC) (Apogee Flow Systems, Hertfordshire, UK), a dedicated FC specially developed for the analysis of nanoparticles (http://www.apogeeflow.com/products.php). Exosomes were left untreated or treated with 0.1 μg of FITC Mouse IgG1 κ Isotype Control (Cat#555748, BD Biosciences, San Jose, CA, USA) was used as background control. Same protocol was followed for the detection of CD44 and CD63 on circulating exosomes and on exosomes from the cell culture supernatants, respectively. For the detection of CD44, after blocking, exosomes were treated with 0.1 μg of FITC mouse anti-human CD44 (Cat#555478, BD Biosciences, San Jose, CA, USA) or with 0.1 μg of FITC Mouse IgG2b κ Isotype Control (Cat#555742, BD Biosciences, San Jose, CA, USA) for 45 min at 4 °C, followed by dilution to 500 μL with PBS. Similarly, for the detection of CD63, exosomes were incubated with 0.025 μg of FITC mouse anti-human CD63 (Cat#557288, BD Biosciences, San Jose, CA, USA) or with 0.025 μg of FITC Mouse IgG1 κ Isotype Control (Cat#555748, BD Biosciences, San Jose, CA, USA) for the same time period followed by dilution to 500 μL with PBS and analysis on Apogee MFC.

The reference ApogeeMix beads (Cat# 1493), which are composed of an aqueous mixture of 110 nm and 500 nm green fluorescent latex beads, have refractive index ɳ = 1.59, and non-fluorescent silica (Si) beads with diameters 180 nm, 240 nm, 300 nm, 590 nm, 880 nm and 1300 nm diameter which have a refractive index ɳ = 1.43, (http://www.apogeeflow.com/products.php), were used to assess the performance of Apogee MFC, and to compare the size distribution of the exosomes. The PBS was run as a background control.

The reference beads and exosomes samples were run following two different settings in Apogee MFC. i) Regular default settings: sample flow rate 0.75 μl/min (total: 130 μl), numerical value set for Laser 405-LALS was 28, numerical value and voltage set for Laser 488-Gre were 26 and 525 V, respectively. ii) High-threshold settings (minimizes the background noise): sample flow rate 1.5 μl/min (total: 130 μl), numerical values set for Thresholds & Lasers 405-SALS, 405-MALS, 405-LALS were 1, 31, 67, respectively; numerical value and voltage set for Laser 488-Gre were 1 and 560 V, respectively.

### Western Blot Analysis

Cells and exosomes were lysed using lysis buffer. Protein lysates of exosomes (5 μg) were run on 4–20% Mini-PROTEIN TGX gel (Bio-Rad, Hercules, California, USA) and transferred to PVDF membrane. The blots were incubated separately either with mouse monoclonal anti-human CD63 antibody (Cat# ab8219, Abcam, Cambridge, MA, USA) at a dilution of 1:10000, mouse monoclonal anti-human CD81 (Cat# NB100–65805, Novus Biologicals, Littleton, CO, USA) at a dilution of 15:10000, rabbit polyclonal anti-human LAMP2B (Cat#ab18529, Abcam, Cambridge, MA, USA) at a dilution of 1:5000, rabbit polyclonal anti-human Grp94 (Cat# 2104P, Cell Signaling Technology, Danvers, MA, USA) at a dilution of 5:10000, or with mouse monoclonal anti-human β-actin (Cat# ab8224, Abcam, Cambridge, MA, USA) at a dilution of 2.5:10000 (in TBS buffer containing 2% BSA) at room temperature for 1 h followed by washing with TBS buffer. The blots were incubated with secondary antibody (horseradish peroxidase, HRP-conjugated goat anti-mouse (Cat# W402B) or goat anti-rabbit IgG (W401B) from Promega, Madison, WI, USA) at a dilution of 1:10000 (2% Milk containing TBS buffer) for 1 h at room temperature. The blots were treated with the ECL kit according to the user manual, developed on X-ray film and finally observed using Konica Minolta SRX-101A Medical Film Processor (Konica Minolta Medical & Graphic Inc., (Shanghai, China).

### Enzyme-linked Immunosorbent Assay (ELISA) for CD47

The level of CD47 in the exosomes isolated from serum samples were determined using a DuoSet^®^ ELISA assay kit for human CD47 (R&D Systems, Minneapolis, MN, USA; Cat# DY4670–05) according to the manufacturer’s instruction. Briefly, the flat-bottom polystyrene 96-well microplates were incubated and coated with the capture antibody overnight at room temperature. On the next day, the plate was washed, blocked with reagent diluent for 1 h at room temperature and washed again. The exosome samples (20 μg protein equivalent amount in 100 μl) or standards were then added for 2 h incubation at room temperature and washed prior to the secondary detection antibody incubation for 2 h at room temperature. After extensive washing, the wells were incubated with Sterptavidin-HRP for 20 min, after washing with the substrate solution for 20 min at room temperature in the dark. After adding the Stop solution, the optical density was measured spectrophotometrically at 450 nm and 560 nm using a microplate reader (GloMax^®^-Multi Detection System, Promega Corporation, Madison, WI, USA). The concentration of CD47 in the samples was determined based on the standard calibration curve.

### Statistical Analysis

Statistical analysis was done by following Unpaired Student’s t-test, Differences among the means were considered to be statistically significant at a p value of P < 0.05 and P < 0.01.

## Additional Information

**How to cite this article**: Kibria, G. *et al*. A rapid, automated surface protein profiling of single circulating exosomes in human blood. *Sci. Rep*. **6**, 36502; doi: 10.1038/srep36502 (2016).

**Publisher’s note:** Springer Nature remains neutral with regard to jurisdictional claims in published maps and institutional affiliations.

## Supplementary Material

Supplementary Information
