# Supplementary material for: A rapid, automated surface protein profiling of single circulating exosomes in human blood
Source: Sci Rep. 2016 Nov 7;6:36502. doi: 10.1038/srep36502 (PMC5098148; doi:10.1038/srep36502)
Supplement: Supplementary Information [file srep36502-s1.pdf]

Supplementary information

A rapid, automated surface protein profiling of single circulating exosomes in human blood

Golam Kibria[1], Erika K. Ramos1, Katelyn E. Lee[1], Sarah Bedoyan[1], Simo Huang[1], Ravand Samaeekia[2], Jaffre J. Athman[1], Clifford V. Harding[1], Jan Lötvall[3], Lyndsay Harris[4], Cheryl L. Thompson[5#], Huiping Liu[1,6,7,8#]

[1]Department of Pathology, School of Medicine, Case Western Reserve University, Cleveland, Ohio, USA. [2]The Institute Molecular Engineering, the University of Chicago, Chicago, IL, USA. [3]Krefting Research Centre, Department of Internal Medicine and Clinical Nutrition, University of Gothenburg, Gothenburg, Sweden. [4]Department of Medicine, [5]Department of Nutrition, School of Medicine, Case Western Reserve University, Cleveland, Ohio, USA. [6]The Case Comprehensive Cancer Center, [7]The National Center for Regenerative Medicine, Case Western Reserve University, Cleveland, OH 44106. [8]Department of Pharmacology, Feinberg School of Medicine, Northwestern University, Chicago, IL 60611

Figure S1

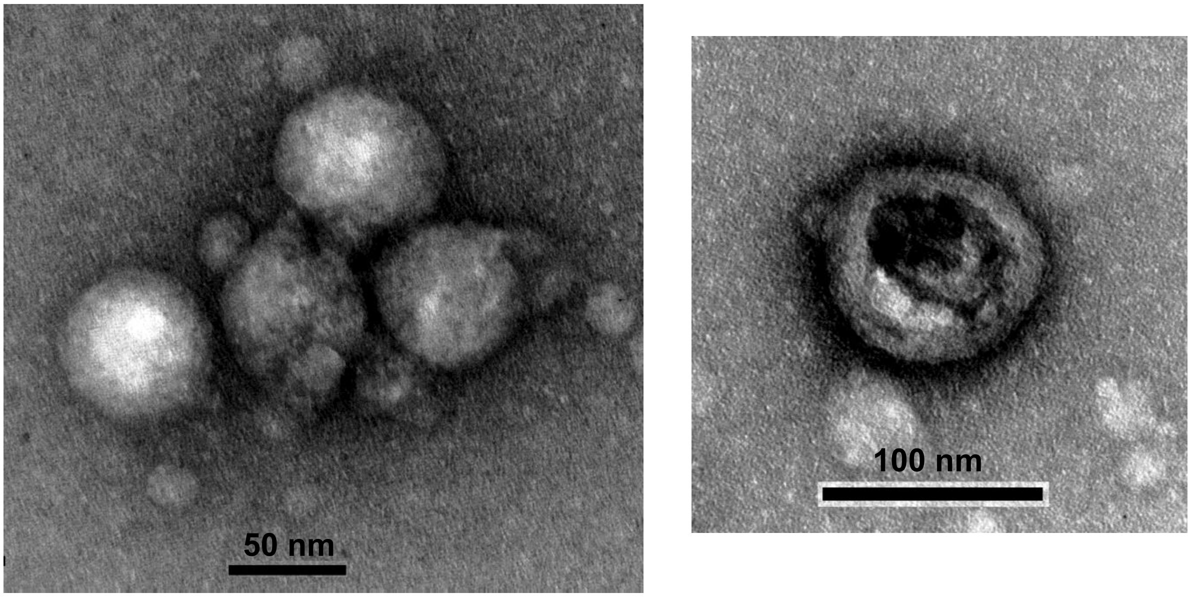

Supplementary Figure S1. The exosome-isolation kit-purified exosomes from human serum (healthy) under Transmission Electron Microscopy (TEM). Exosomes were isolated from the serum after depletion of cell debris and apoptotic bodies per manufacturerôs manual.

Figure S2

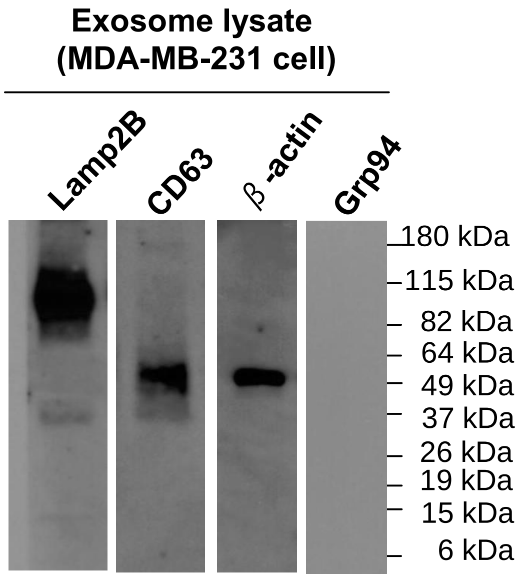

Supplementary Figure S2. Evaluation of the expression of exosomal markers on cell derived exosomes. Western blots of 5 μg exosome lysates for the exosomal markers LAMP2B (~100 kDa) and CD63 (~55kDa). Grp94 (~100 kDa) and β -actin (~42 kDa) serves as a negative control and loading control, respectively.

Figure S3

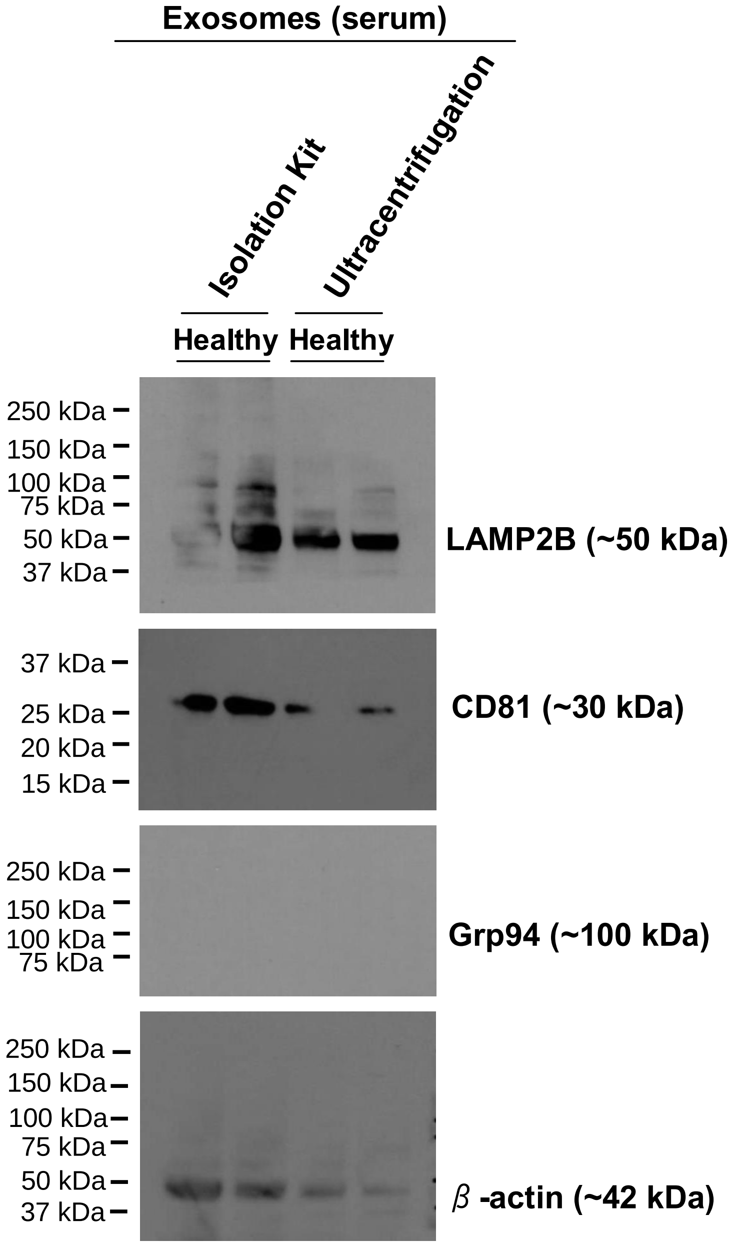

Supplementary Figure S3. Immunoblot comparison of the expression of markers on exosomes isolated from human serum (healthy) by differential ultracentrifugation and by the exosome-isolation kit. Exosomal markers LAMP2B (~50 kDa) and CD81 (~30 kDa) were detected in exosomes (5 μg lysates). Grp94 (~100 kDa) and β-actin (~42 kDa) serve as a negative control and loading control, respectively.

Figure S4

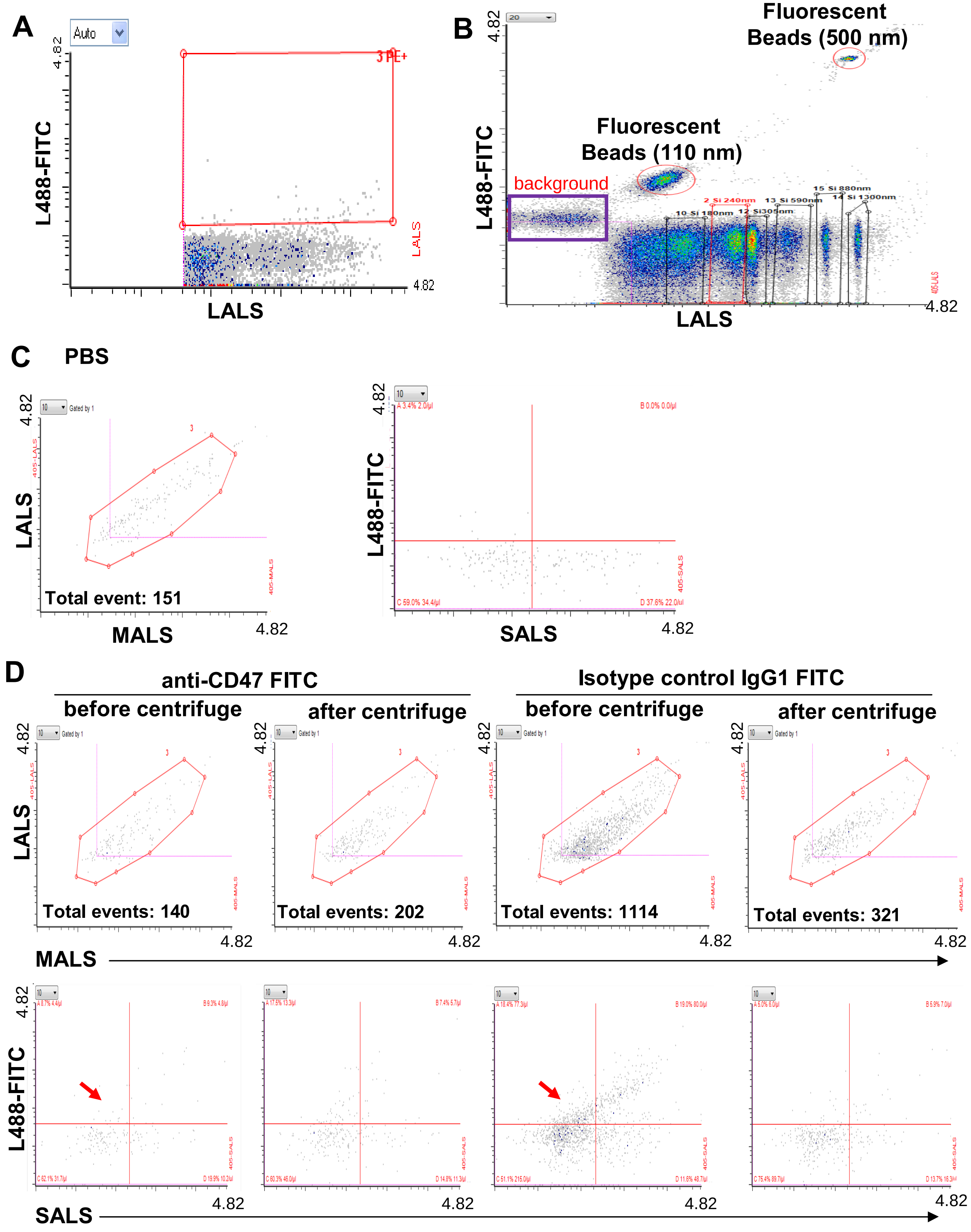

A
Auto
L488-FITC
LALS
4.82
3 PE+
B
Fluorescent Beads (500 nm)
Fluorescent Beads (110 nm)
background
L488-FITC
LALS
4.82
10 Si 180nm
2 Si 240nm
12 Si305nm
13 Si 590nm
15 Si 880nm
14 Si 1300nm
C
PBS
LALS
MALS
Total event: 151
L488-FITC
SALS
4.82
D
anti-CD47 FITC
Isotype control IgG1 FITC
before centrifuge
after centrifuge
before centrifuge
after centrifuge
LALS
Total events: 140
Total events: 202
Total events: 1114
Total events: 321
MALS
L488-FITC
SALS
4.82

Supplementary Figure S4. Exosome flow analysis optimizations.

A-B. Background particles shown in PBS (A) and the reference beads solution (B) at the default setting of A50 MFC. The reference ApogeeMix beads is an aqueous mixture of 110 nm and 500 nm green fluorescent latex beads with refractive index (RI) $\eta=1.59$, and non-fluorescent silica (Si) beads with 180-1300 nm diameter and RI $\eta=1.43$. The RI of Si beads is closer to the RI of biological particles (RI of cells and EVs is 1.4).

C-D. Analyses of PBS and antibodies using A50 MFC. **C.** PBS, used for exosome sample preparations, was run at high-threshold setting. Minimal particles in PBS were observed in multiple light scatter and fluorescence L488 channels. **D.** Cytogram of FITC-CD47 antibody as well as Isotope control FITC-IgG1 before and after centrifugation. Before staining exosomes, antibody bulk solutions were centrifuged at 14000 x g for 1h at 4 ^{o}C to remove particles or precipitates.

Figure S5

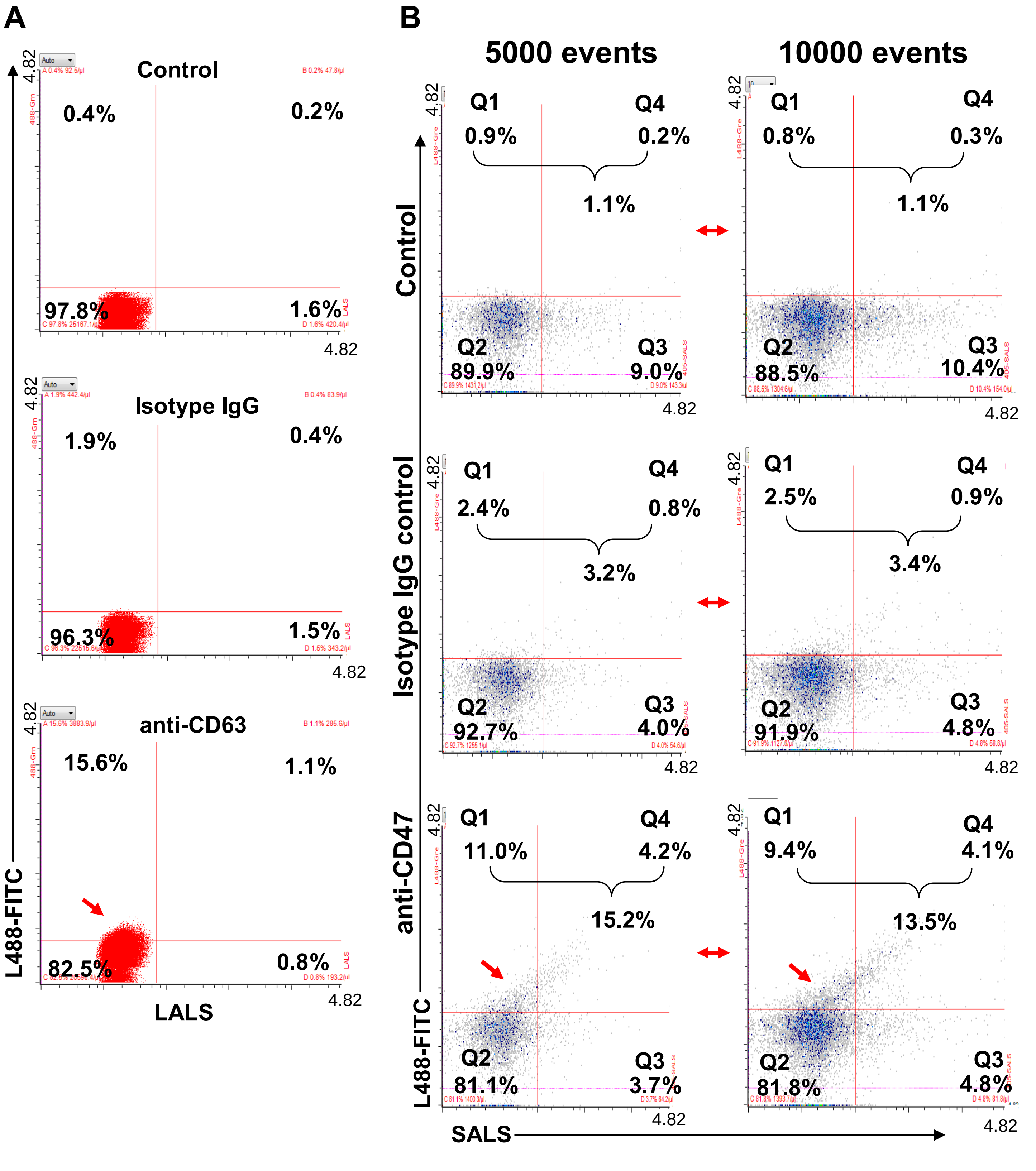

A
Control
0.4%
0.2%
97.8%
1.6%
Isotype IgG
1.9%
0.4%
96.3%
1.5%
anti-CD63
15.6%
1.1%
82.5%
0.8%
4.82
L488-FITC
LALS
B
5000 events
10000 events
Control
Q1 0.9%
Q4 0.2%
1.1%
Q2 89.9%
Q3 9.0%
Q1 0.8%
Q4 0.3%
1.1%
Q2 88.5%
Q3 10.4%
Isotype IgG control
Q1 2.4%
Q4 0.8%
3.2%
Q2 92.7%
Q3 4.0%
Q1 2.5%
Q4 0.9%
3.4%
Q2 91.9%
Q3 4.8%
anti-CD47
Q1 11.0%
Q4 4.2%
15.2%
Q2 81.1%
Q3 3.7%
Q1 9.4%
Q4 4.1%
13.5%
Q2 81.8%
Q3 4.8%
L488-FITC
SALS

Supplementary Figure S5. MFC analyses of exosomal CD63 and CD47

A. Detection of CD63 expression on exosomes derived from MDA-MB-231 cells by A50 MFC. Exosomes were stained with the FITC-conjugated mouse anti-human CD63 antibody, followed by dilution with PBS and then detected under Apogee MFC. FITC Isotype IgG staining or unstained were used as background controls. The expression of CD63 was detected (red arrow) in 15.6% exosomes.

B. Evaluation of the effect of total events counted on CD47 detection on circulating exosomes. Cytograms showing the expression of CD47 in 5000 and 10000 counted exosomes isolated from the blood of healthy control. Exosomes were stained with FITC-CD47 antibody, isotype control FITC-IgG1, or unstained (control). Between the analyses with 5000 and 10000 counted events, similar % of CD47+ exosomes were observed in the upper two quadrants (Q1+Q4).

Figure S6

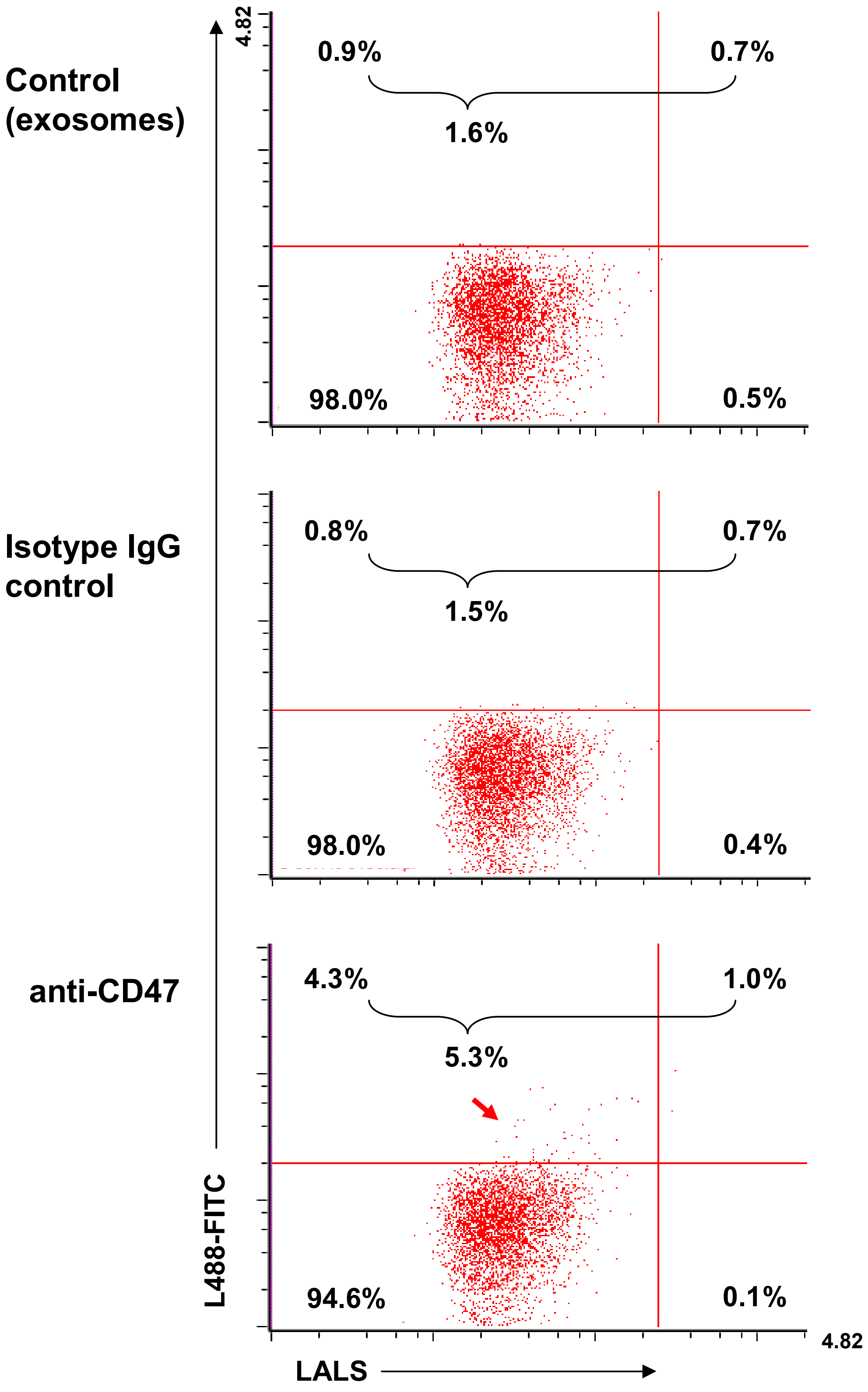

Supplementary Figure S6. Detection of CD47 expression on exosomes isolated from human serum (healthy) by using the exosome-isolation kit. Exosomes were stained with FITC-CD47 antibody or isotype control FITC-IgG1, or unstained (control) followed by detection under Apogee MFC.

Figure S7

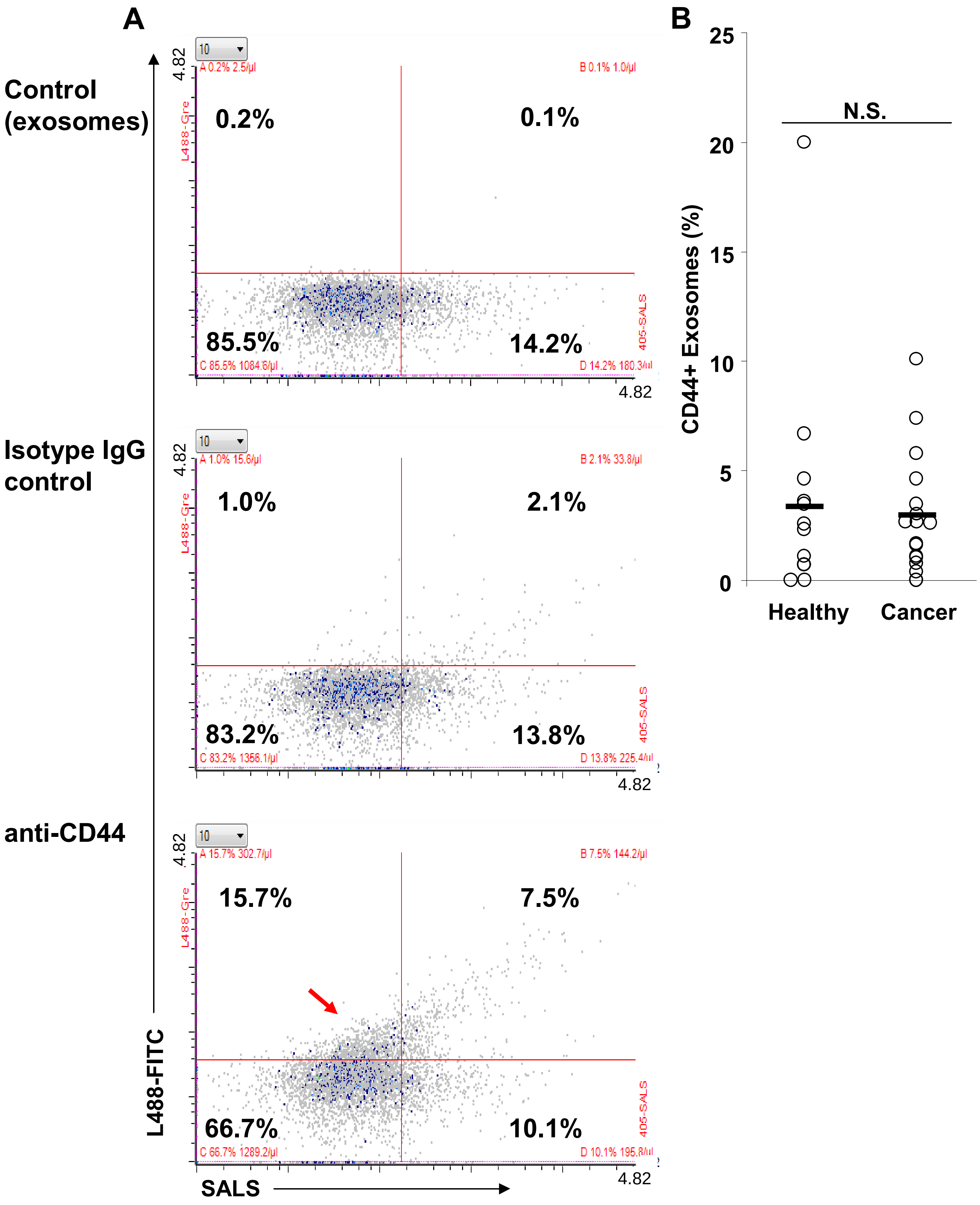
A
Control (exosomes)
Isotype IgG control
anti-CD44
L488-FITC
SALS
4.82
0.2%
0.1%
85.5%
14.2%
1.0%
2.1%
83.2%
13.8%
15.7%
7.5%
66.7%
10.1%
B
CD44+ Exosomes (%)
N.S.
25
20
15
10
5
0
Healthy
Cancer

Supplementary Figure S7. Detection of CD44 on circulating exosomes by MFC.

A. Cytograms showing the expression of CD44 on exosomes isolated from the blood of a healthy control. Exosomes were stained with either FITC-CD44 antibody, isotype control FITC-IgG, or unstained (control). Compared to controls, about 20% CD44 positive exosomes were detected (red arrow).

B. Comparison of the expression of CD44 in circulating exosomes isolated from the blood of fifteen healthy controls and twenty breast cancer patients. Statistical analysis was done by Unpaired Studentôs t-test. N.S.: non-significant.

Supplementary Table S1. Clinical information of the female breast cancer patients and healthy controls (female) used in this study.

Parameters	Breast Cancer Patients	Healthy Control
Total population	60	60
Age (years)	48±10	50±7
Sex	Female	Female
Tumor size (cm)	4.7±2.4	-
Her2+	43	-
ER+	38	-
PR+	28	-
Treatment status (prior to blood drawn)		
Untreated	39	-
Resection	15	-
Hormonal therapy	1*	-
Mastectomy	3	-
Unknown	3	-

HER2+: HER2 positive; ER+: Estrogen receptor positive; PR+: Progesterone receptor positive; * indicates patient received hormonal therapy in addition to resection.
